# Supplementary figures and images for: A machine learning enhanced EMS mutagenesis probability map for efficient identification of causal mutations in Caenorhabditis elegans
Source: PLoS Genet. 2024 Aug 26;20(8):e1011377. doi: 10.1371/journal.pgen.1011377 (PMC11379379; doi:10.1371/journal.pgen.1011377)

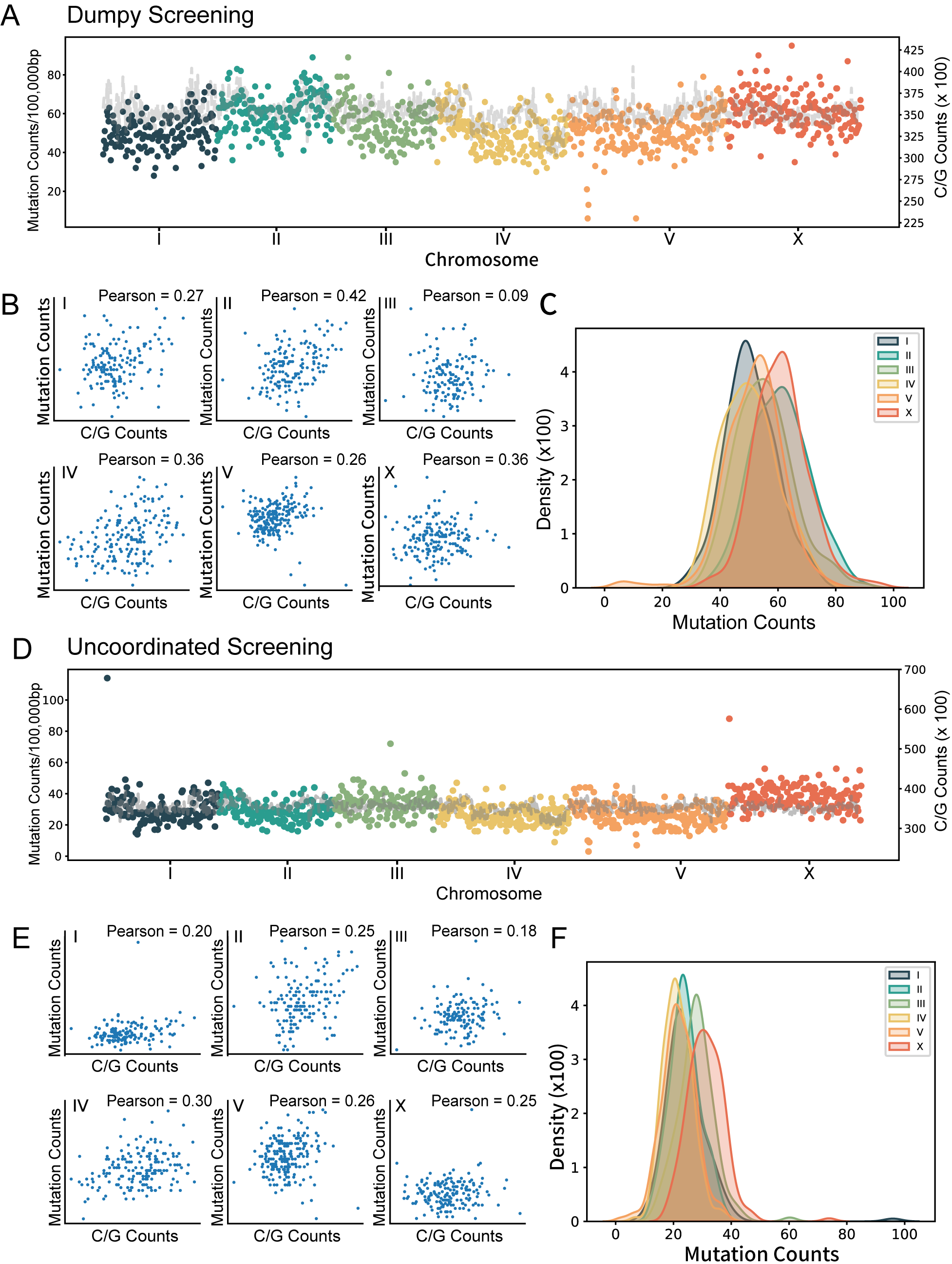

Supplement: S1 Fig — A) Scatter and line plots representing mutations in the Dumpy screening dataset (n = 240). The Scatter plot shows the mutation number on each chromosome. Each dot represents the number of mutated bases within every 100,000 bp counted from the first base pair of each chromosome. The Line plot represents the number of ‘C/G’ base pairs in each 100,000 bp regions. B) Scatter plot representing the relationship between ‘C/G’ base pair contents and the number of mutations from the Uncoordinated screening data of each chromosome. Each dot represents the number of mutated bases within every 100,000 bp counted from the first base pair of each chromosome. Pearson correlation was used to evaluate the association between them. C) Kernel density estimation plot of the number of mutations on each chromosome in uncoordinated screening dataset. D) Scatter and line plots representing mutations in the Uncoordinated screening dataset (n = 118). The Scatter plot shows the mutation number on each chromosome. Each dot represents the number of mutated bases within every 100,000 bp counted from the first base pair of each chromosome. The Line plot represents the number of ‘C/G’ base pairs in each 100,000 bp regions. E) Scatter plot representing the relationship between ‘C/G’ base pair contents and the number of mutations from the dumpy screening data of each chromosome. Each dot represents the number of mutated bases within every 100,000 bp counted from the first base pair of each chromosome. Pearson correlation was used to evaluate the association between them. F) Kernel density estimation plot of the number of mutations on each chromosome in dumpy screening dataset. (TIF) [file pgen.1011377.s001.tif]

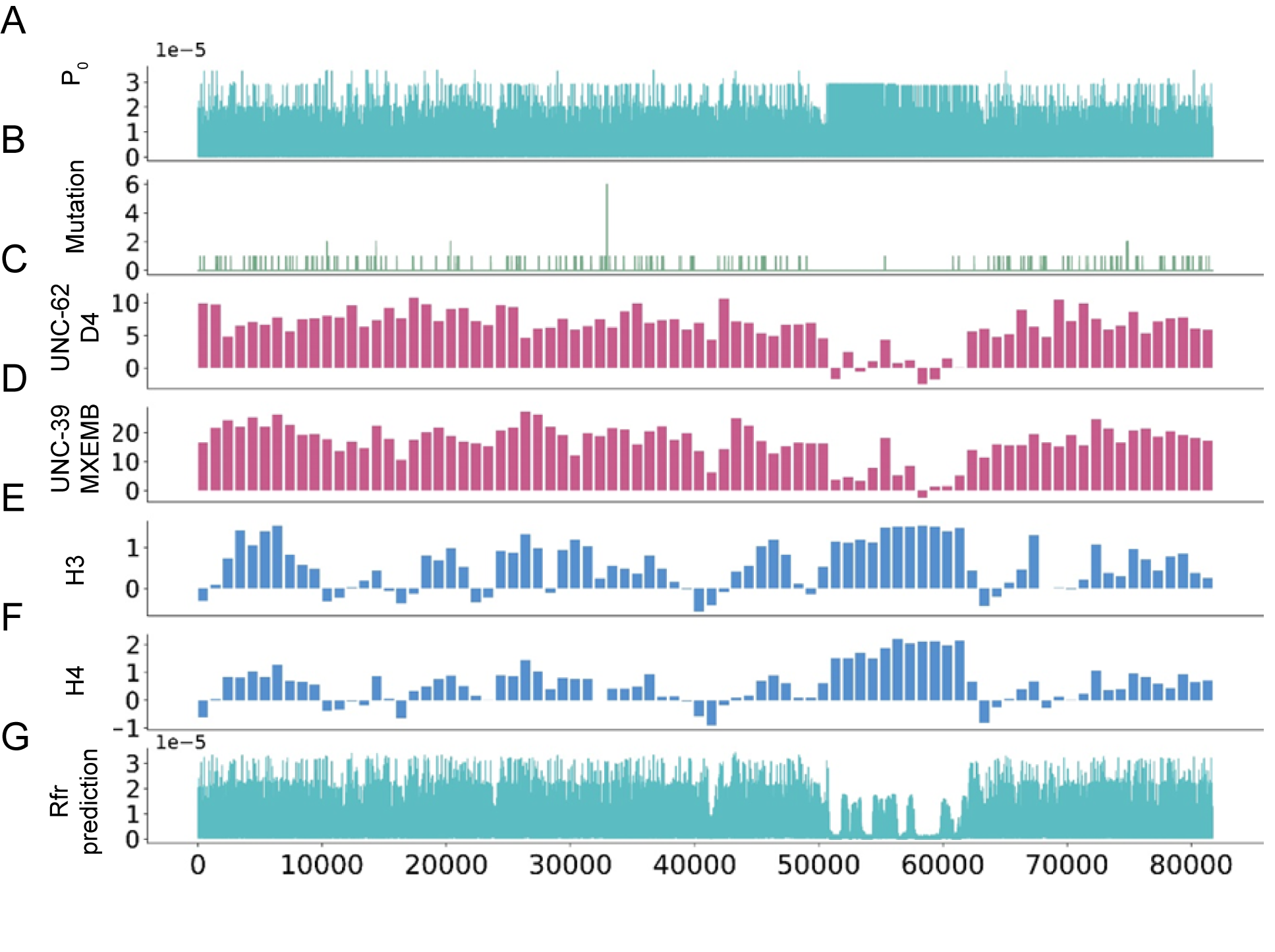

Supplement: S2 Fig — Take the MMP data and DNA-binding protein features on Chr V:6120909~6202632 (ttn-1) as an example. A) Line plots showing the prediction made from the Flanking sequence preferences of EMS mutagenesis (P0). B) The mutations observed in the MMP dataset. C) Raw ChIP-chip signal data of UNC-62 binding in young adult worms. D) Raw ChIP-chip signal data of UNC-39 binding in worm embryo. E) Raw ChIP-seq signal data of histone H3 in L3 larval worms. F) Raw ChIP-seq signal data of histone H4 in L3 larval worms. G) Line plots showing the prediction made by a Random Forest regressor trained by DNA-binding protein data. (TIF) [file pgen.1011377.s002.tif]

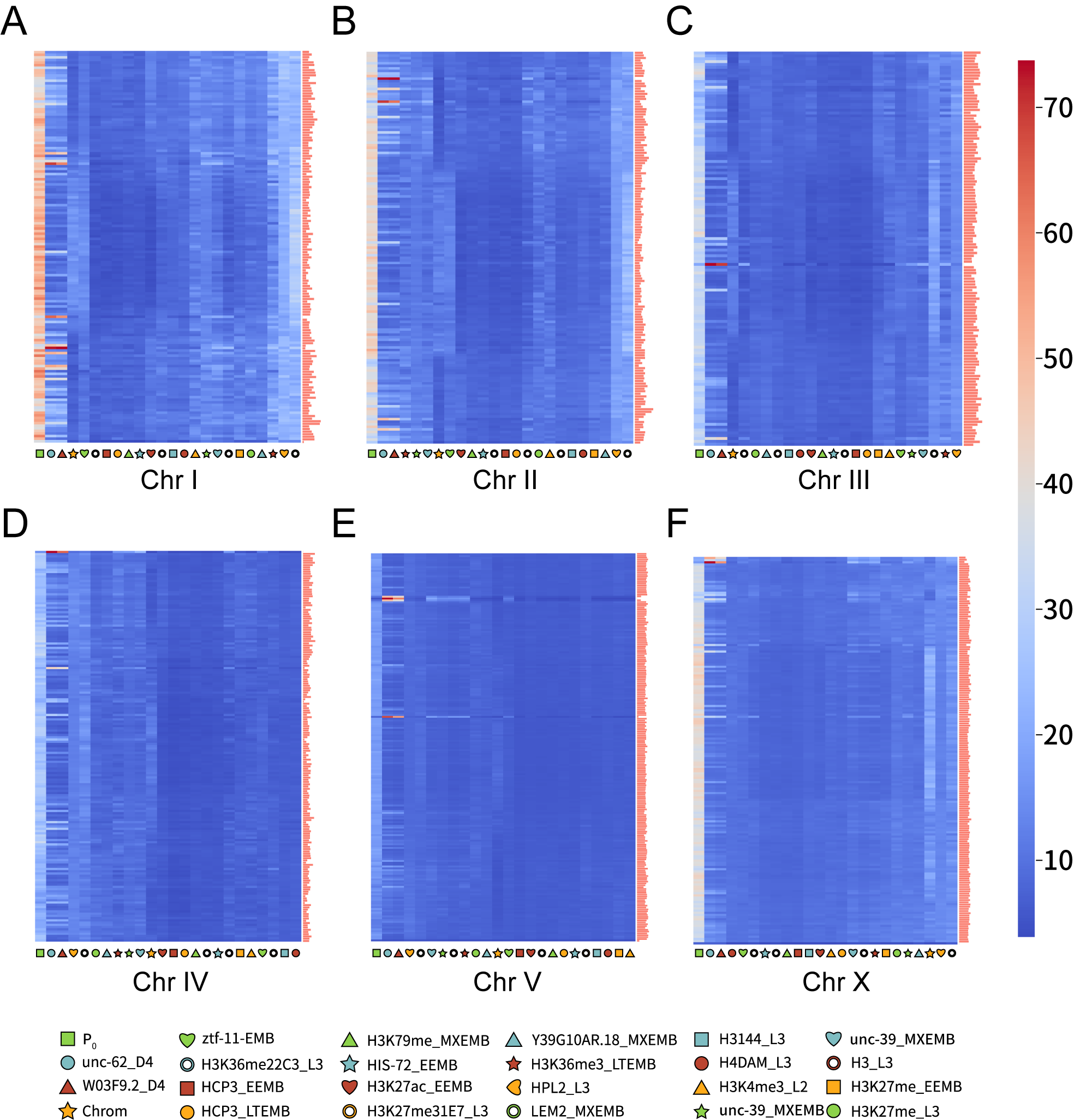

Supplement: S3 Fig — A-F) Permutation importance (see Materials and Methods) of each feature used to model the Random Forest regressor. The importance is shown in the heatmap alongside the chromosome. Mutation rate is normalized to 0~1 and is shown in the bar plot attaching to the right side of the heatmap. (TIF) [file pgen.1011377.s003.tif]
