# Supplementary material for: A machine learning enhanced EMS mutagenesis probability map for efficient identification of causal mutations in Caenorhabditis elegans
Source: PLoS Genet. 2024 Aug 26;20(8):e1011377. doi: 10.1371/journal.pgen.1011377 (PMC11379379; doi:10.1371/journal.pgen.1011377)
Supplement: S1 Table — (DOCX) [file pgen.1011377.s004.docx]

**S1 Tabel**

**Features for Random Forest training**

| **Feature Name** | **Information** | **Resource** |
| --- | --- | --- |
| Chrom | Which chromosome the base belongs to | Reference genome WS235 |
| Code | 𝑃_0_ or ‘pentabase bias’ | Calculated with the MMP dataset |
| HPL2_L3 | ChIP-ChIP of L3 worm targeting heterochromatin protein HPL2; strain: N2 | GSE40947 |
| LEM2_MXEMB | ChIP-ChIP of mixed-stage embryo targeting nuclear envelope protein LEM2; strain: N2 | GSM562786 |
| unc-62_D4 | ChIP-seq of day4 young adult worm targeting transcription factor unc-62; strain: OP600 | GSM942047 |
| W03F9.2_D4 | ChIP-seq of day4 young adult worm targeting transcription factor W03F9.2; strain: OP215 | GSM729307 |
| H3K36me22C3_L3 | ChIP-ChIP of early embryo targeting K36trimethylated histone H3; strain: N2 | GSE22719 |
| HCP3_EEMB | ChIP-ChIP of early embryo targeting holocentric chromosome binding protein HCP3; strain: N2 | GSM1255286 |
| H3K27ac_EEMB | ChIP-ChIP of early embryo targeting K27 acetylated histone H3; strain: N2 | GSM562779 |
| HCP3_LTEMB | ChIP-ChIP of late embryo targeting holocentric chromosome binding protein HCP3; strain: N2 | GSM1255289 |
| H3K79me_MXEMB | ChIP-ChIP of mixed-stage embryo K79 trimethylated histone H3; strain: N2 | GSM562779 |
| HIS-72_EEMB | DNA tilling array of mixed-stage embryo; strain: JJ2061 | modEncode 5326 |
| H3_L3 | ChIP-ChIP of L3 worm targeting histone H3; strain: N2 | GSM1005484 |
| H3K27me31E7_L3 | ChIP-ChIP of L3 worm targeting K27trimethylated histone H3; strain: N2 | GSM562735 |
| H3144_L3 | ChIP-ChIP of L3 worm targeting histone H3; strain: N2 | GSM562745 |
| H4DAM_L3 | ChIP-ChIP of L3 worm targeting histone H4; strain: N2 | GSM624427 |
| H3K36me3_LTEMB | ChIP-ChIP of late embryo targeting K36trimethylated histone H3; strain: N2 | GSM811293 |
| unc-62_EEMB | ChIP-seq of mixed-stage embryo targeting transcription factor unc-62; strain: OP600 | GSE48731 |
| unc-39_EEMB | ChIP-seq of mixed-stage embryo targeting transcription factor unc-39; strain: OP186 | GSE46775 |
| Y39G10AR.18_MXEMB | ChIP-ChIP of mixed-stage embryo targeting transcription factor Y39G10AR.18; strain: N2 | GSM811254 |
| H3K27me_L3 | ChIP-ChIP of L3 worm targeting K27 trimethylated histone H3; strain: N2 | GSM562735 |
| H3K27me_EEMB | ChIP-ChIP of early embryo targeting K27 trimethylated histone H3; strain: N2 | GSM811255 |
| H3K4me3_L2 | ChIP-ChIP of L2 worm targeting K4 trimethylated histone H3; strain: N2 | GSM811289 |
| ztf-11-EMB | ChIP-seq of mixed-stage embryo targeting zinc finger putative transcription factor ztf-11; strain: OP236 | GSE48746 |
