# Supplementary material for: A machine learning enhanced EMS mutagenesis probability map for efficient identification of causal mutations in Caenorhabditis elegans
Source: PLoS Genet. 2024 Aug 26;20(8):e1011377. doi: 10.1371/journal.pgen.1011377 (PMC11379379; doi:10.1371/journal.pgen.1011377)
Supplement: S2 Table — (DOCX) [file pgen.1011377.s005.docx]

**S2 Tabel**

***C. elegans* Strains in this study**

| **Strain Name** | **Genotype** | **Method** |
| --- | --- | --- |
| N2 | *wild-type* | N.A |
| cas22599 | *OSM-3 ^E251K^* | Microinjection |
| cas23441 | [OSM-3 ^E251K^; K04F10.2 ^Arg506*^] | Microinjection |
