# Supplementary material for: A machine learning enhanced EMS mutagenesis probability map for efficient identification of causal mutations in Caenorhabditis elegans
Source: PLoS Genet. 2024 Aug 26;20(8):e1011377. doi: 10.1371/journal.pgen.1011377 (PMC11379379; doi:10.1371/journal.pgen.1011377)
Supplement: Attachment — Submitted filename: comments to authors.docx [file pgen.1011377.s007.docx]

The manuscript by Guo et al. described an analysis of SNPs from a collection of several hundreds of *C. elegans* mutants obtained from EMS mutagenesis and generated an EMS mutagenesis probability map for the C. elegans genome. The authors then used this map to identify several causal mutations from a suppressor screen of a C. elegans ciliary kinesin mutant *osm-3*. The findings that the EMS-induced mutations in the C. elegans genome are not uniformly distributed across the genome and the probability of EMS mutation on specific genome region depends on adjacent sequence and local chromatin status are quite interesting. However, it is unclear about the generality of using the EMS probability map reported in this manuscript to identify causal genes from various forward genetic screens. Specific comments are as follows.

Major concerns:

1. The identification of causal mutations using the mutagenesis probability map described in this manuscript seems to work only with screens that obtain multiple alleles from the same set of genes. Especially, in the suppressor screen of *osm-3* with 38 independent suppressors described here, there are 10, 12, and 16 different alleles for three genes. I’m not convinced that the same approach (without mapping) will still be productive to identify causal genes in genetic screens that only recover one or two alleles of each relevant gene. However, I am open to being persuaded. For difficult forward genetic screens (e.g. manual fluorescence-based screen under high power microscopy), it will take a lot more screen time to get more alleles than just map the available alleles.
2. One of the main points of the manuscript is to apply the new method to identify causal genes. It will be helpful to provide more details about the experimental details. For instance, how to use the EMS mutagenesis probability map to identify casual mutations in a new screen if the reader wants to use it.
3. In line with point2, the discussion section would discuss the pros and cons of the method in the context of current mapping and cloning methods (e.g. mapping with CB4856 or the SSM method) and highlight the conditions where the reported method will be more productive to identify causal genes.
4. The mutation counts/100,000bp from the three different sets of EMS screens (MMP project, Uncoordinated screen and Dumpy screen) are quite different: ~200-300 for the MMP set (Figure 1A and C), 40-60 for uncoordinated screen (Figure S1A and C) and 20-40 for the Dumpy screen (Figure S1D and F). With such large variants on the mutate rate from different screens, how effectively the same EMS mutagenesis probability map reported can be used to pinpoint the causal genes.

Minor concerns:

1. It will be helpful to explain the figure legend better for readers to understand. For example, figure 1B, what does each pot present?
2. The manuscript needs to be proofread thoroughly. Some examples (but not limited to these): line 158; line 258, (ref); line 369, define the Dyf phenotype; lines 469 470: italicize the gene names (and other places in the manuscript); line 624: (47), fix the style of the reference; line 689: strain name (and other places in the manuscript), gene name and genotype need to be corrected according to the standard nomenclature ([Nomenclature - WormBase : Nematode Information Resource](https://wormbase.org/about/userguide/nomenclature#835l206fjcbi7ak4e9mh1dg--10)).
