# Supplementary material for: A machine learning enhanced EMS mutagenesis probability map for efficient identification of causal mutations in Caenorhabditis elegans
Source: PLoS Genet. 2024 Aug 26;20(8):e1011377. doi: 10.1371/journal.pgen.1011377 (PMC11379379; doi:10.1371/journal.pgen.1011377)
Supplement: Attachment — Submitted filename: 20240718 PLOS Genetics response.docx [file pgen.1011377.s008.docx]

**RESPONSE TO THE REVIEWERS**

Reviewer Comments:

Reviewer #1:

Chemical-mutagenesis forward-genetic screening is a powerful tool in the realm of genetic screening. Forward genetic techniques offer advantages over reverse genetic techniques (RNAi and CRISPR/Cas9) in that it can reveal novel genes and lead to the discovery of unexpected connections and mechanisms. However, identifying the responsible mutation for a given phenotype is laborious and can require a significant time investment.

The authors of this study outline the development of a machine learning tool that can be used to identify causative mutations generated through EMS chemical mutagenesis. They show that the distribution of EMS-induced mutations is non-uniform throughout the genome, both within and across chromosomes; specific nucleotide sequences and different chromatin statuses are biased towards different mutation frequency. This is a key observation that is used to train the Random Forest regressor (Rfr) machine learning tool which can predict what genes give rise to an observed phenotype following mutagenesis. The Rfr model is used to identify the suppressor gene K04F10.2 after EMS-mutagenesis, which can restore ciliary function in OSM-3 mutant C. elegans. Taken together, the conclusions reached in the study are very promising. However, I have a number of concerns about the figures and the explanation of key takeaways as outlined below.

We thank the Reviewer for the positive comments to our study.

Major points

1) Introduction. The discussion of genetic screening techniques is adequate. Given that the study intersects with the field of machine learning, there should also be a priming outline of machine learning practices that already exist in the field. Add more background for strictly biological researchers who may not be familiar with machine learning techniques and probability mapping.

An expanded background on machine learning practices would benefit readers, particularly those with a strictly biological background. Therefore, we have revised the introduction to include additional context on machine learning techniques and their applications in genetic screening. The background information has been integrated into our introduction section, specifically on page 7 line 120:

“…Machine learning, widely employed as a statistical method, serves as a potent tool for uncovering hidden relationships between variables, constructing predictive models from data, and identifying informative patterns within datasets

(Greener, et al., 2022). It proves particularly valuable when datasets are too complex for straightforward human analysis, enabling the capture of underlying relationships between variables. Recent advancements in this field have successfully linked phenotypes to potential causal variants using sequencing data (Mountjoy, et al., 2021) or structural information (Cheng, et al., 2023). Existing tools primarily predict variant impact based on sequence conservation or clinical datasets, such as SIFT (Ng and Henikoff, 2003) or SnpEff (Cingolani, et al., 2012). However, in genetic screening involving highly effective mutagens, pathogenic alleles may not always be causal, and the genotype-phenotype relationship can be complicated by genetic backgrounds. Moreover, numerous genes whose functions remain poorly understood may not cause disease but are still worthy of investigation. Therefore, a new model for genetic screening is essential to identify causal mutations independently of their known functions and predict the significance of mutated genes in chemical screening experiments….”

2) Line 201. Formulation of P0 should also be inserted into the main text since many future panels rely on an understanding of this calculation.

We have now inserted the formulation at first time ‘P0’ was mentioned in this article in on page 9 line 194:

“…Utilizing this information, the base mutation frequency can be determined by calculating the mutation rate of each pattern in all the mutation events in the Million Mutation Project. Hereafter we refer to this base mutation frequency as $P_{0}$, which is determined by:$P_{0}=\frac{C^{'}}{C_{0}}$. $C^{'}$represents the count of a specific five-base pattern underwent mutation in the MMP dataset and $C_{0}$ is the number of the same pattern in the reference genome…”

3) Figure 3E/F. It is stated that a distinctive histone binding pattern is not seen in the ‘silent region’ of figure 3 as is the case in the *ttn-1* gene. Yet there does seem to be a slight increase in histone binding activity in this region compared to the entire chromosome sequence depicted, particularly in H3. The text does not elaborate on panels E/F specifically, and the conclusion that this pattern is “not observed” is not entirely convincing.

We have rewritten the text for a more precise description about the ChIP-seq dataset on page 13 line 241:

“… a distinct histone binding pattern is evident in this region (SI Appendix, Fig. S2E, F). While histone binding shows a slight increase across the preceding 200 kb 'silent region' on chromosome V (Fig. 3 E, F), particularly in H3, the profile of transcription factor binding provides a clearer boundary of this area. This suggests that transcription factor binding may be a more effective feature for capturing the relationship between abnormal mutation frequency and DNA-binding proteins in this area ….”

4) Figure 3E/F. There are conflicting results for how histone modification affects mutation frequency in the ‘silent region’ versus the ttn-1 gene. Is it known how “alternative chromatin states” (line 226) affect mutagenesis? Are there experiments that can elucidate the binding patterns at play here? Figure 3 as a whole requires a more detailed explanation with greater emphasis on the rhetorical flow of logic.

We appreciate the reviewer's suggestion. In our manuscript, the concept of 'alternative chromatin states' aligns with 'chromatin accessibility,' which assesses whether a DNA region is accessible to transcription factors and other nuclear macromolecules. However, we refrain from asserting that these chromatin states directly determine EMS accessibility due to their inherent complexities. Methodologies to determine EMS accessibility to the genome is currently unavailable. Consequently, establishing a causal relationship among ChIP-seq data, mutation frequency, and chromatin states poses a challenge. Nevertheless, alterations in histone and transcription factor binding patterns may influence EMS effectiveness in specific genomic regions. Rather than inferring causality between these features and mutation frequency, we acknowledge leveraging correlations for predictive purposes. We have addressed this issue in the revised manuscript, specifically on page 27 line 521:

“…The Random Forest model elucidated the relationship between various DNA-binding protein patterns and mutation frequency. DNA-binding proteins such as histones and transcription factors are commonly employed to assess the accessibility of genomic DNA, from which the topological arrangement of nucleosomes can be inferred (Klemm, et al., 2019). However, this relationship becomes more intricate due to the challenge of precisely uncovering how DNA-binding protein patterns affect EMS effectiveness. For example, the occupancy of transcription factors on chromatin may reduce accessibility to EMS, while regions lacking ChIP-seq signals likely indicate a tightly coiled DNA topology with nucleosomes, making them less accessible to other molecules. The machine learning model established the relationship between EMS mutation frequency and these features by leveraging these correlations, resulting in a comprehensive model capable of handling diverse patterns. Further experiments are necessary to decipher these patterns and gain a deeper understanding of the actual topology and conditions of these regions.…”

5) Figure 5E/F, lines 436-440. The results given in these panels are unclear and appear incongruous with the written text. Did OSM-3 E251K; K04F10.2 double mutants receive the Scarlet-tagged protein or wild type K04F10.2? Is the succeeding condition with Scarlet protein meant to show a diminished capacity of K04F10.2-tagged Scarlet to rescue OSM-3?

We appreciate the reviewer for pointing out this issue. In our genetic screen, we utilized GFP-tagged CHE-3, an IFT-dynein heavy chain, as a marker for cilia to demonstrate sensory cilia length. K04F10.2 was identified as a suppressor for the OSM-3 E251K mutation using machine learning. To validate this prediction, we employed CRISPR-Cas9-based genome editing to knockout the K04F10.2 gene in the OSM-3 E251K strain. The resulting double mutant animals recapitulated the ciliary phenotype observed in the double mutant isolated from our EMS screen. Additionally, introducing Scarlet-tagged wild-type genomic DNA of K04F10.2 into the double mutant resulted in ciliary defects resembling those of the single OSM-3 E251K mutation. These results collectively confirm K04F10.2 as a causal gene. In accordance with Reviewer #2's suggestion, we have renamed K04F10.2 as *jbts-26*.

We have now changed the description about the rescue experiment on page 24 line 468:

“…While OSM-3 E251K;K04F10.2 double mutants restored ciliary length to 7.54±0.60 μm, introducing Scarlet-tagged wild-type genomic DNA of K04F10.2 into the double mutant reduced ciliary length to 6.22±0.81 μm, similar to that of the OSM-3 E251K single mutant. These results indicate that K04F10.2 mutations act as suppressors for OSM-3 E251K. Henceforth, we designate this gene as *jbts-26* due to its inferred role in primary cilia. …”

Minor points

1) Line 456. typo in “laborintensive”

We have corrected this typo.

2) Lines 229-241. Nearly identical paragraphs repeated back-to-back.

We have deleted the repeated part.

3) Line 257. Missing reference for WormBase.

We have added the reference in page 15 line 277.

4) Figure 5 legend. Font inconsistency.

We have corrected this inconsistency.

5) Figure S1. Misplaced label in panel B.

We have corrected the misplaced label. We thank the Reviewer for pointing out these issues.

Reviewer #2:

The manuscript by Guo et al presents an innovative and potentially game-changing strategy for chemically-induced forward genetic screening. In the past, mutagenesis-based genetic screens were standard in model organism fields. With technological advances including bioinformatics and genomic engineering, reverse genetic strategies have become the standard to ascertain gene function. Due to the difficulty in identifying causal lesions in mutagenesis screens and ease of knock-down/knockout strategies, forward screens are falling out of favor. However, as authors point out, the power of forward genetic screens is their unbiased nature, their ability to identify novel mutations that may be otherwise missed, and their power to reveal unknown biology. Here, authors use a strategy that capitalizes on the numerous community generated resources (WormBase, MillionMutations project, CHIP-seq, modENCODE) to identify causal mutations in WGS in two proof-of-principle EMS-based screens. In addition to this, authors show that mutation frequency is influenced by nearby sequences and chromatin status. This manuscript will appeal to the readership of PLOS Genetics, with some modification.

As written, the reader needs to be expert in C. elegans, in statistics/machine learning, and in cilia biology. This reviewer is versed in two, but not machine learning and struggled with this section of the manuscript. For example, it would help to more thoroughly explain how the Random Forest Regressor works and why this model was chosen. The same would be true for the non-worm or non-cilia reader in those section. Authors must make the manuscript accessible to the broad readership of PLOS Genetics, so that the impact and usefulness of this powerful strategy can be appreciated and employed by model organism geneticists.

We appreciate that the Reviewer is supportive to our study.

To improve readability, we have revised the text and incorporated additional background information on the decision tree model. This aims to facilitate a clearer understanding of the Random Forest model on page 15, line 282.

“…A decision tree is a simple yet powerful prediction method (Krzywinski and Altman, 2017). It splits data into subsets in order to create groups with similar values of the target feature. Therefore, the predictor can make predictions from new observations with the relationship it learned from the already exist data. Random Forests are a combination of tree predictors (Breiman, 2001). Each tree is trained on a randomly selected subset of the data and features, capturing slightly different information, and reducing the risk of overfitting, thus improving overall performance.

We developed a regression model using Random Forest Regressor. The underlying hypothesis is that, despite the sparsity of mutation events in the Million Mutation Project (200-350 mutations per 100,000 bp, Fig. 1A), the mutation probability for base pairs with the same 'property' is well-represented in the dataset. The random forest algorithm's task is to categorize base pairs into different groups based on DNA binding protein patterns and flanking sequences, enabling accurate prediction of each group's average mutation expectation (Fig. 4A). Nucleotides with similar flanking sequences and DNA-binding protein attributes are grouped into the same output node of each tree. The mean mutagenesis frequency for that node then serves as the predicted mutagenesis frequency for any nucleotide with similar characteristics assigned to that node during predictions.

To create our model, we used a dataset containing 24 DNA-binding protein datasets from modENCODE, which includes information about histone binding, modifications, transcription factor interactions, and other epigenetic modifications (SI Appendix, Table S1). We constructed 600 decision trees, each based on the mentioned methodology, with each tree trained on a randomly chosen subset of up to 16 protein features. To create our model, we used a dataset containing 24 DNA-binding protein datasets from modENCODE, which includes information about histone binding, modifications, transcription factor interactions, and other epigenetic modifications (SI Appendix, Table S1). We constructed 600 decision trees, each trained on a randomly chosen subset of up to 16 protein features, based on the methodology described above…”

Second, while the data is convincing that this strategy works, how can this be applied in a practical sense? It would be great if authors could work with WormBase/Alliance of Genome Resources to develop a user-friendly interface. I realize this is beyond the scope of this manuscript, but hope this is a future direction.

We appreciate the reviewer's suggestion. Indeed, we plan to organize a user-friendly graphical user interface (GUI) to benefit the worm community.

Currently, users can utilize our model by downloading all the necessary model data and running a Python script locally. All required files, including a detailed README file, can be downloaded from [young55775/EMSForest-A-Machine-Learning-Enhanced-EMS-Mutagenesis-Probability-Map (github.com)](https://github.com/young55775/EMSForest-A-Machine-Learning-Enhanced-EMS-Mutagenesis-Probability-Map).

We have added this information to the Code availability section on page 35 line 711:

“…To facilitate the use of our model, we provide all necessary model data and a Python script that can be run locally. These resources, along with a detailed README file, can be downloaded from [young55775/EMSForest-A-Machine-Learning-Enhanced-EMS-Mutagenesis-Probability-Map (github.com)](https://github.com/young55775/EMSForest-A-Machine-Learning-Enhanced-EMS-Mutagenesis-Probability-Map). In the future, we will work with WormBase/Alliance of Genome Resources to develop a user-friendly interface to benefit the community…”

A few minor things:

Lines 195, 197: typo isare

We have corrected the typo.

Lines 230-242 are redundant/garbled

We have deleted the repeated word.

Throughout manuscript: check C. elegans nomenclature for italicizing gene names

line 369 define “back door” (example of needing to be a cilia/kinesin aficionado)

suggestion – request K04F10.2 be named *jbts-26*

We have named this gene as *jbts-26* following the suggestion.

We have modified the description about the OSM-3 E251K mutation to make it easy to be understood in page 20 line 391:

“…The E253K mutation in KIF1A is hypothesized to affect the flexibility of switch I in the motor domain…”

line 434 – Fig. 5E

We have corrected it.

lines 465, 468: I think kinesin-II is older nomenclature. Heterotrimeric kinesin-2?

We have now used heterotrimeric kinesin-2.

Reviewer #3:

The manuscript by Guo et al. described an analysis of SNPs from a collection of several hundreds of C. elegans mutants obtained from EMS mutagenesis and generated an EMS mutagenesis probability map for the C. elegans genome. The authors then used this map to identify several causal mutations from a suppressor screen of a C. elegans ciliary kinesin mutant osm-3. The findings that the EMS-induced mutations in the C. elegans genome are not uniformly distributed across the genome and the probability of EMS mutation on specific genome region depends on adjacent sequence and local chromatin status are quite interesting. However, it is unclear about the generality of using the EMS probability map reported in this manuscript to identify causal genes from various forward genetic screens. Specific comments are as follows.

We appreciate the Reviewer's insightful comments. We acknowledge that our EMS probability map cannot be universally applicable, particularly in challenging scenarios such as manual fluorescence-based screens under high-power microscopy, as the Reviewer pointed out. However, many types of forward genetic screens can efficiently generate tens of mutant alleles with manageable workload. In our revised manuscript, we have deliberated on the applicability and versatility of our approach, underscoring its practical utility in diverse experimental contexts.

Major concerns:

The identification of causal mutations using the mutagenesis probability map described in this manuscript seems to work only with screens that obtain multiple alleles from the same set of genes. Especially, in the suppressor screen of osm-3 with 38 independent suppressors described here, there are 10, 12, and 16 different alleles for three genes. I’m not convinced that the same approach (without mapping) will still be productive to identify causal genes in genetic screens that only recover one or two alleles of each relevant gene. However, I am open to being persuaded. For difficult forward genetic screens (e.g. manual fluorescence-based screen under high power microscopy), it will take a lot more screen time to get more alleles than just map the available alleles.

Our model is primarily based on statistical methods that achieve optimal accuracy when multiple alleles are obtained from screening experiments. It may not be efficient in the scenarios, as pointed out by the Reviewer. Some screening experiments are inherently difficult, making it hard to acquire the target phenotype. For instance, manual fluorescence-based screens under high-power microscopy fall into this category. In such cases, we recommend using the sibling subtraction method instead of our tool. Machine learning approaches rely on statistical effects to achieve desired accuracy, assuming a sufficient number of candidates are screened—typically, we recommend 30 or more samples per experiment. Our tool was originally designed for large-scale causal gene identification. For small datasets, sibling subtraction remains the most reliable method when dealing with only a few alleles.

Given the affordability of whole-genome sequencing (WGS), WGS data are usually accessible prior to sibling subtraction experiments. Analyzing one hundred WGS datasets using the EMS probability map takes less than one minute, and only several seconds for 10 to 20 WGS datasets. Our model significantly enhances the likelihood of identifying potential causal mutations for genes that have low baseline mutation probabilities, irrespective of the number of isolated mutant alleles. Therefore, investing an additional minute in computation could potentially expedite mutant cloning efforts.

While the EMS probability map cannot be universally applied, especially in challenging screens that yield a limited number of mutant alleles, it proves valuable in many forward genetic screens capable of efficiently generating dozens of mutants with manageable effort. Nonetheless, it's crucial to employ multiple complementary approaches to enhance forward genetic screens. Their strength lies in their capacity to uncover novel mutations that might otherwise go unnoticed, and ability to unveil unknown aspects of biology. Thus, the EMS probability map can be a useful addition to the forward genetic toolkit.

In our revised Discussion, we have highlighted the limitations and advantages of our method on page 25, line 493:

“…Our model performs well when multiple alleles per gene are obtained but is less effective when screens yield only one or two alleles per gene. For challenging forward genetic screens such as manual fluorescence-based screens under high-power microscopy, we recommend using the sibling subtraction method. Machine learning approaches rely on statistical effects to achieve desired accuracy, assuming a sufficient number of candidates are screened—typically, we recommend 30 or more samples per experiment. Our tool was originally designed for large-scale causal gene identification. For small datasets, sibling subtraction remains the most reliable method due to its manageable workload when dealing with only a few alleles.

Given the affordability of whole-genome sequencing (WGS), WGS data are usually available before sibling subtraction experiments. Analyzing one hundred WGS datasets using the EMS probability map takes less than one minute, and only several seconds for 10 to 20 WGS datasets. Our model significantly enhances the likelihood of identifying potential causal mutations for genes that have low baseline mutation probabilities, irrespective of the number of isolated mutant alleles. Therefore, investing an additional minute in computation could potentially expedite mutant cloning efforts.

While the EMS probability map cannot be universally applied, especially in challenging screens that yield a limited number of mutant alleles, it proves valuable in many forward genetic screens capable of efficiently generating dozens of mutants with manageable effort. Nonetheless, it's crucial to employ multiple complementary approaches to enhance forward genetic screens. Their strength lies in their capacity to uncover novel mutations that might otherwise go unnoticed, and ability to unveil unknown aspects of biology. Thus, the EMS probability map can be a useful addition to the forward genetic toolkit…”

One of the main points of the manuscript is to apply the new method to identify causal genes. It will be helpful to provide more details about the experimental details. For instance, how to use the EMS mutagenesis probability map to identify casual mutations in a new screen if the reader wants to use it.

We appreciate the reviewer's suggestion. Indeed, we plan to organize a user-friendly graphical user interface (GUI) to benefit the worm community.

Currently, users can utilize our model by downloading all the necessary model data and running a Python script locally. All required files, including a detailed README file, can be downloaded from [young55775/EMSForest-A-Machine-Learning-Enhanced-EMS-Mutagenesis-Probability-Map (github.com)](https://github.com/young55775/EMSForest-A-Machine-Learning-Enhanced-EMS-Mutagenesis-Probability-Map).

We have added this information to the Code availability section on page 35 line 711:

“…To facilitate the use of our model, we provide all necessary model data and a Python script that can be run locally. These resources, along with a detailed README file, can be downloaded from [young55775/EMSForest-A-Machine-Learning-Enhanced-EMS-Mutagenesis-Probability-Map (github.com)](https://github.com/young55775/EMSForest-A-Machine-Learning-Enhanced-EMS-Mutagenesis-Probability-Map). In the future, we will work with WormBase/Alliance of Genome Resources to develop a user-friendly interface to benefit the community….”

In line with point2, the discussion section would discuss the pros and cons of the method in the context of current mapping and cloning methods (e.g. mapping with CB4856 or the SSM method) and highlight the conditions where the reported method will be more productive to identify causal genes.

We agree with the reviewer to explain the specific condition where the reported method will be more productive. We have provided the answer in our reply to point1 above.

The mutation counts/100,000bp from the three different sets of EMS screens (MMP project, Uncoordinated screen and Dumpy screen) are quite different: ~200-300 for the MMP set (Figure 1A and C), 40-60 for uncoordinated screen (Figure S1A and C) and 20-40 for the Dumpy screen (Figure S1D and F). With such large variants on the mutate rate from different screens, how effectively the same EMS mutagenesis probability map reported can be used to pinpoint the causal genes.

We apologize for any confusion that may have arisen due to the data presentation, which did not explicitly specify the varying numbers of EMS-mutagenized worms across the different datasets. Below is detailed information on mutation counts from various screens:

MMP Project:

737 WGS datasets of EMS mutagenized worms yield a total of 265,169 mutations, averaging 360 mutations per worm. This results in approximately 265 mutations per 100,000 base pairs (bp) across all 737 worms. Calculating per worm gives approximately 0.36 mutations per 100,000 bp.

DPY Dataset:

240 EMS mutagenized worms yield 65,171 mutations, averaging 272 mutations per worm. This results in approximately 65 mutations per 100,000 bp across all 240 worms. Calculating per worm gives approximately 0.27 mutations per 100,000 bp.

UNC Dataset:

118 EMS mutagenized worms yield 34,730 mutations, averaging 293 mutations per worm. This results in approximately 35 mutations per 100,000 bp across all 118 worms. Calculating per worm gives approximately 0.30 mutations per 100,000 bp.

While the MMP project generates a slightly higher mutation rate (360 mutations per worm) compared to our DPY (272 mutations per worm) or UNC (293 mutations per worm) screens, all mutation rates fall within a similar range. We have provided the information in the revised Discussion on page 27, line 536:

“…We summarize the mutation counts from various datasets: In the MMP project, 737 whole-genome sequencing datasets of EMS mutagenized worms revealed a total of 265,169 mutations, averaging 360 mutations per worm, or approximately 0.36 mutations per 100,000 base pairs (bp). The DPY dataset, consisting of 240 worms, showed 65,171 mutations, averaging 272 mutations per worm, or approximately 0.27 mutations per 100,000 bp. Lastly, the UNC dataset, with 118 worms, yielded 34,730 mutations, averaging 293 mutations per worm, or approximately 0.30 mutations per 100,000 bp. While the MMP project generates a slightly higher mutation rate (360 mutations per worm) compared to our DPY (272 mutations per worm) or UNC (293 mutations per worm) screens, all mutation rates fall within a similar range….”

To apply our pipeline on screening datasets with different EMS effectiveness, we have normalized the variation in the mutation rates from each dataset:

“…We took the mutation frequency of the Million Mutation Project as a baseline. We denote the mutagen effectiveness of the MMP strains as $\alpha_{0}$ and the efficiency in another experiment as $\alpha_{1}$, the random forest regression model takes the form of: $P=\alpha_{0}f(x,P_{0})$, where x represents the DNA-binding protein features used by the model to make predictions and adjust the initial prediction of $P_{0}$. This model can be applied to another dataset as: ${P'}_{seq}=\alpha_{1}\sum_{i\in seq} f\left( x_{i},P_{0} \right)=\frac{\bar{N}_{1}}{\bar{N}_{0}}\alpha_{0}\sum_{i\in seq} f\left( x_{i},P_{0} \right)$, Here, $\bar{N}_{0}$ and $\bar{N}_{1}$ represent the average number of mutation events that occurred in the MMP strains and the batch to be analyzed, respectively. In this way, this model can be used on screening with altered EMS effectiveness…”

We have now provided the above information in the Method section of the revised manuscript on page 28, line 657. We appreciate that the Reviewer for pointing out this issue.

Minor concerns:

It will be helpful to explain the figure legend better for readers to understand. For example, figure 1B, what does each pot present?

We have now provided additional details in figure legends.

The manuscript needs to be proofread thoroughly. Some examples (but not limited to these):

line 158;

We have corrected the typo.

line 258, (ref);

we have added the reference.

line 369, define the Dyf phenotype;

We have added the definition of Dyf, Dpy, and Unc phenotype where it first be mentioned in the article.

lines 469 470: italicize the gene names (and other places in the manuscript);

We have checked the manuscript and corrected the gene names.

line 624: (47), fix the style of the reference;

We have corrected the style of the reference.

line 689: strain name (and other places in the manuscript), gene name and genotype need to be corrected according to the standard nomenclature ([Nomenclature - WormBase : Nematode Information Resource](https://wormbase.org/about/userguide/nomenclature#835l206fjcbi7ak4e9mh1dg--10)).

We have corrected the strain names, gene names and genotype. We thank the Reviewers for pointing out these issues.
